# Biochemical, electrolytic, and cardiovascular evaluations in cats with urethral obstruction

**DOI:** 10.14202/vetworld.2021.2002-2008

**Published:** 2021-08-04

**Authors:** Darlan Henrique Canei, Mariana Elisa Pereira, Maria Natália de Freitas, Yolanda Paim Arruda Trevisan, Carolina Zorzo, Juliano Bortolini, Adriane Jorge Mendonça, Valéria Régia Franco Sousa, Arleana do Bom Parto Ferreira de Almeida

**Affiliations:** 1 Program of Postgraduate in Veterinary Sciences, Faculty of Veterinary Medicine, Federal University of Mato Grosso, Cuiabá – Mato Grosso, Brazil; 2 Scientific Initiation Volunteer (CNPq), Faculty of Veterinary Medicine, Federal University of Mato Grosso, Cuiabá – Mato Grosso, Brazil; 3 Department of Statistics, Federal University of Mato Grosso, Cuiabá - MT, Brazil; 4 Veterinary Hospital, Faculty of Veterinary Medicine, Federal University of Mato Grosso, Cuiabá – Mato Grosso, Brazil

**Keywords:** arrhythmia, feline lower urinary tract disease, hyperkalemia, troponin I cardiac

## Abstract

**Background and Aim:**

Urethral obstruction (UO) is a common condition in feline medicine. Severe acid-base and electrolyte disorders promote relevant electrocardiographic changes in these animals. Cardiac biomarkers such as cardiac troponin I have been shown to be useful in identifying cats with myocardial disease, but it has not been investigated whether UO leads to myocardial damages. This study aimed to evaluate biochemical changes, electrocardiographic findings, troponin I measurements, and electrolyte disturbances for 7 days in cats with UO.

**Materials and Methods:**

This follow-up prospective study included 33 cats diagnosed with UO for 7 days. For all cats, clinical examination, serum biochemistry, electrolyte analyses, blood pressure, and electrocardiography were performed. Cardiac troponin I was measured in the serum in 16 cats at 3 different times.

**Results:**

The mean age of the feline population was 1.83±1.58 years (mean±standard deviation). Creatinine, urea, blood urea nitrogen, glucose, phosphorus, base excess, bicarbonate, and serum potassium decreased significantly (p≤0.05), while ionic calcium and blood pH increased significantly (p≤0.05) at different times. Electrocardiographic abnormalities were observed in 21/33 (63.63%) of the felines on admission day. The electrocardiographic abnormalities were no longer observed on the subsequent days. Only one feline showed changes in troponin I cardiac concentrations.

**Conclusion:**

This study suggests the sum and severity of electrolyte abnormalities aggravate the clinical and cardiovascular status of these patients. However, cTnI, blood pressure, and heart rate within the reference range do not exclude the presence of major cardiovascular and metabolic abnormalities. The hyperglycemia in felines with UO appears to be associated with decreased renal clearance, which may reflect the severity of hyperkalemia and azotemia. The metabolic and cardiovascular changes of these felines are minimized by the establishment of appropriate intensive care; however, cardiac and blood gas monitoring is essential to assess the severity of the disease.

## Introduction

Urethral obstruction (UO) in male cats due to feline lower urinary tract disease (FLUTD) is encountered in veterinary medicine and usually leads to acute loss of renal function and, in turn, to acid-base and electrolyte imbalances [1,2]. The initial clinical signs include hematuria, polyuria, and stranguria [3]. The incidence of this disease varies from 1% to 6.3% [4-6]. The diagnosis is based on anamnesis and physical examination; distended urinary bladder is the main clinical finding [1]. Due to the distension of the urinary bladder and its increased intravesical pressure, urine returns to the kidneys, causing increased intratubular pressure, decreasing the glomerular filtration rate, thus affecting tubular concentration capacity, especially reabsorption, and water-sodium regulation, and potassium excretion, leading to uremia, acidosis, and hyperkalemia [7]. Hyperkalemia may result in changes in atrioventricular membrane potential, leading to arrhythmias, which may progress to ventricular fibrillation [8]. In 2003, Lee and Dobratz [9] evaluated 223 cats with UO, characterizing clinical, electrolytic, acid-base, and renal alterations. It was observed that most cats with UO exhibited mild changes in blood gases and electrolytes, being relatively stable, and only 12% of the cats evaluated had life-threatening metabolic disorders, but no serial follow-up was performed; therefore, it did not describe the evolution of laboratory alterations in these patients.

Troponins I and T (cTnI and cTnT) have been described as high sensitivity and specificity cardiac markers in myocardial injuries in humans [10]. Troponin I is the most sensitive isoform for myocardial wall injuries [11-14]. Myocardial injury has been documented in many non-cardiac diseases, especially in patients with heart disease, inflammatory, and shock [13,15,16]. Patients with or without myocardial injury have similar clinical presentations, so the measurement of cardiac troponin is necessary to discover the involvement of myocardial injury in patients with critical illness [17,18]. Myocardial injury is not clinically recognized; however, its presence is associated with an increased risk of morbidity and mortality [16]. Dogs and humans, with evidence of myocardial injury, have a 4-fold higher mortality rate than those with normal troponin concentrations [16,19,20]. It has been shown that 41% of cats assessed with UO developed heart murmur during hospitalization, and arrhythmias cardiac disorders were noted in 55% of hospitalized cats [21]. According to the authors, felines who presented changes in cardiac auscultation were 4.5 times more likely to develop effusions, reinforcing the importance of researching cardiac injuries in cats with obstructive FLUTD. It has been characterized that non-cardiac diseases can lead to myocardial injury in humans [22-24], dogs [17,25-27], and cats [28]; however, these studies were limited to showing high concentrations of troponins on time, without further cardiac and neither the monitoring of the lesion, the relevance of these findings remains unclear.

The present study performed the serial analysis at different times (before and after clearance) of laboratory, metabolic, and cardiological characteristics of male cats with UO.

## Materials and Methods

### Ethical approval

The study complied with the ethical principles for animal experimentation according to the Ethical Principles in Animal Experimentation (Brazilian College of Animal Experimentation, COBEA) and was approved by the Animal Use Ethics Committee (CEUA – UFMT nº 23108.928840/2018-73). All tutors were informed about the procedures performed and allowed the inclusion of their animals after providing informed written consent.

### Study period and location

This study was conducted from May 2018 to December 2019 at the Small Animal Medical Clinic of the Veterinary Hospital of the Federal University of Mato Grosso (HOVET-UFMT), Cuiabá, Mato Grosso, Brazil.

### Cats

A prospective 7 days follow-up cohort study was performed. Felines of different weights and breeds with UO, younger than 7 years old, presented to the emergency services were selected. The diagnosis of UO was based on a clinical history of stranguria, pain on abdominal palpation (hypogastric region), and bladder distension.

### Clinical analysis

Samples of venous blood (5 mL), collected on the day of admission (D1) and on day 2 (D2) and day 7 (D7) (post-obstruction), were placed in tubes without anticoagulant. Samples were centrifuged at 5000 rpm for 5 min to measure serum creatinine, blood urea nitrogen (BUN), total plasma proteins, albumin, globulin, aspartate aminotransferase, alkaline phosphatase and total calcium, phosphorus, and magnesium electrolyte levels using an automated biochemical analyzer (Wiener Lab Group, Santa Fe, Argentina). Whole blood glucose was measured by a portable glucometer (Accu-Chek Active, Roche, Basel, Switzerland). Venous blood gas analysis was performed using a lithium heparin-containing syringe (A-Line, BD, Franklin Lakes, NJ, USA) immediately after collection using a blood gas analyzer (Cobas b121, Roche, Basel, Switzerland) for pH, base excess (BE), partial pressure of oxygen, partial pressure of carbon dioxide, bicarbonate (HCO_3_) concentration, and potassium, sodium, and chloride levels. Serum cTnI concentrations were determined by immunoenzymatic assay (ELISA) using purified specific cTnI, containing feline monoclonal antibodies, using the feline ELISA Kit troponin I (Genorise, USA). The samples were kept frozen at –80°C, analyzed in duplicate, according to the manufacturer’s recommendations.

### Cardiac assessment

Thorough cardiopulmonary auscultation was performed by an experienced operator to identify murmurs, arrhythmias, crackles, and/or effusions in all animals. Standard 6-lead electrocardiogram (ECG) (leads I, II, III, aVR, aVL, and aVF) was recorded for ≥3 min in all felines using a computerized ECG monitor (Teb Eletrônica Brasileira, São Paulo, Brazil) at a speed of 50 mm/s calibrated to 10 mm/mV. ECGs were evaluated for changes in T-wave and ST-segment amplitude, arrhythmias and conduction disorders, and heart rate and rhythm [29].

Non-invasive blood pressure measurements were recorded using an automated oscillometric method (petMAP, Ramsey Medical Inc., Tampa, FL, USA). Five consecutive readings were obtained, and the minimum and maximum values were discarded; the average values were calculated according to the American College of Veterinary Internal Medicine consensus [3].

### Anesthesia and urethral catheterization

The same anesthetic protocol was performed in all study patients using propofol (5 mg/kg) and lidocaine (4 mg/kg) sacrococcygeal epidural block (0.1 mg/kg) according to the American Association of Feline Practitioners anesthesia guidelines [30]. Catheterization was performed using surgical antisepsis and a penile massage and 20-gauge or 22-gauge catheter, with polyvinyl urethral tube fixation (Kendall Sovereign, Kendall Inc., USA) for a period of 48 h [31]. The animals were subjected, as part of the treatment, to fluid therapy with Ringer’s lactate (2 mL/kg/h) [32], prazosin (0.5 mg/cat) [33], tramadol (2 mg/kg), and vitamin complex.

### Statistical analysis

Data were analyzed using online statistical software (R version 3.5.0, R Foundation for Statistical Computing, Vienna, Austria). Variables are expressed as minimum, maximum, median, and standard deviation. In addition to descriptive analysis, Kruskal–Wallis non-parametric tests were performed for equality between all medians, and the Conover and Iman test for comparison between medians. Pearson correlation was used to compare variables according to established criteria [34]. For all analyses, a significance level of 5% was established.

## Results

Thirty-three felines were initially included in the study on D1; however, four animals were excluded on D2 (two deaths and two referred for surgery after unsuccessful attempts to pass a urinary catheter), and three on D7 (discharge from the hospital after urinary catheter removal) and without follow-up. The age of the animals ranged from 5 months to 5 years, with mean of 1.83±1.58 years. Most felines (84.84%) were not a defined breed, while 15.15% had a racial definition (four Siamese and one Persian). Hypovolemic shock because of dehydration was observed in three of the 33 cats studied, severe dehydration in 5, moderate and mild dehydration in 16 and 9, respectively. Among the clinical signs, vocalization and apathy were most observed, 88.46% and 84.61%, respectively, while stupor was found in 69.23%. Emesis and diarrhea were verified in 57.69% and 46.15%, respectively, and seizures were observed in 3 of 33 (9.09%) at the time of initial care. Descriptive statistics and the results of univariate analysis for physical examination, blood pressure, serum biochemistry, blood gas analysis, and ECG data at different observation times are summarized in Tables-1 and 2.

**Table 1:** Descriptive statistics and univariate analysis of the clinical and biochemical of cats with UO of the study at times D1, D2, and D7.

Variables	Reference interval	D1	D2	D7	p-value
		
		M±SD	M±SD	M±SD	
		
		(n=33)	(n=29)	(n=26)	
Clinical					
HR (bpm)	140-220	175±44.17	170±37.68	195±34.85	0.034*
RR (mrm)	20-40	20±15.84	18±1.96	20±3.13	0.256
BT (°C)	37-39.2	37.7±1.47	38.2±0.61	38.25±0.39	<0.001*
DBP (mmHg)	88-104	103±27.98	96±21.64	90±20.51	0.176
MBP (mmHg)	107-123	130±29.19	115±22.98	112±16.72	0.057
SBP (mmHg)	123-141	172±36.80	147±30.07	150±17.09	0.043*
Biochemical					
Creatinine (mg/dL)	0.8-1.8	10.3±6.98	2.85±4.84	1.2±0.83	<0.001*
BUN (mg/dL)	14-36	161.47±103.68	90.07±87.50	22.16±11.87	<0.001*
Albumin (g/dL)	2.1-3.3	3.2±0.41	3.1±0.26	3.1±0.23	0,499
ALT (UI/L)	26-43	52±35.21	48±29.26	41±30.03	0.228
AF (UI/L)	25-93	81±83.28	45±36.82	45.5±21.63	0.007*
Globulin (g/dL)	2.6-5.1	3.5±0.77	3.5±0.62	3.55±7.26	0.892
Total protein (g/dL)	5.4-7.8	6.7±0.91	6.5±0.70	6.65±0.86	0.673
Glucose (mg/dL)	70-110	124±55.12	97±29.03	90.5±19.67	<0.001*
Total calcium (mg/dL)	6.2-10.2	8.3±6.65	8.9±0.89	9.65±0.71	<0.001*
Phosphorus (mg/dL)	4.5-8.1	12.5±8.91	8.9±4.48	5.45±1.13	<0.001*
Magnesium (mg/dL)	1.9-2.6	3.3±0.63	3.2±0.67	2.5±0.24	<0.001*

M=Median; DP=Standard deviation; n=Total of animals; D1=Day 1; D2=Day 2; D7=Day 7; HB=Heart rate; RR=Respiratory rate; BT=Body temperature; DBP=Diastolic blood pressure; MBP=Mean blood pressure; SBP=Systolic blood pressure; ALT=Alanine aminotransferase; AF=Alkaline phosphatase;

* statistics difference. BUN=Blood urea nitrogen

**Table 2:** Descriptive statistics and univariate analysis of feline blood gas analysis and ECG with UO at times D1, D2, and D7.

Variables	Reference interval	D1	D2	D7	p-value
		
		M±SD	M±SD	M±SD	
		
		(n=33)	(n=29)	(n=26)	
Blood gas					
pH	7.3-7.4	7.27±0.09	7.29±0.08	7.33±0.06	0.003*
pO_2_	40-50	42.9±7.07	36.4±7.39	34.8±4.89	<0.001*
pCO_2_	32.7-44.7	35.2±6.29	41.7±6.78	35.3±8.67	0.002*
BE (mmol/L)	−2±3	−9.6±5.01	−7.8±4.35	−5.5±3.22	0.004*
HCO_3_ (mmol/L)	18-23.2	15.5±3.34	18.9±3.60	19.8±3.50	0.001*
Potassium (mmol/L)	3.5-5.4	5.97±2.03	4.05±0.88	3.55±0.64	<0.001*
Sodium (mmol/L)	147-156	149.7±8.55	157.9±4.97	163.1±4.58	<0.001*
Chloro (mmol/L)	111-125	115±9.14	124.3±5.67	124.2±6.16	<0.001*
ECG					
P height (mV)	≤0.2	0.07±0.11	0.11±0.09	0.12±0.10	0.039*
P width (s)	≤0.04	0.04±0.04	0.04±0.01	0.04±0.01	0.788
PR interval (s)	0.05-0.09	0.07±0.04	0.06±0.02	0.06±0.01	0.2
R height (mV)	≤0.9	0.33±0.24	0.34±0.21	0.36 ± 0.22	0.368
QRS duration (s)	≤0.04	0.05±0.02	0.04±0.01	0.04±0.01	0.233
ST height (mV)	-	0.03±0.06	0±0.05	0±0.01	<0.001*
QT interval (s)	0.12-0.18	0.19±0.04	0.16±0.16	0.14 ± 0.03	<0.001*
T height (mV)	≤0.3	0.12±0.26	0.09±±0.18	0.12±0.08	0.1869
T width (s)	≤0.07	0.07±0.03	0.05±0.02	0.05±0.02	0.1132

M=Median; SD=Standard deviation; n=Total of animals; D1=Day 1; D2=Day 2; D7=Day 7; BE=Base excess; HCO_3_=Bicarbonate;

* statistics difference. ECG=Electrocardiogram

There was no significant association between mean heart rate and serum potassium concentration; however, 6 (18.18%) animals exhibited bradycardia at the time of initial care with a very strong negative correlation (r=−0.94; p=0.068) with serum potassium concentration. There was a strong negative correlation (r=−0.64; p<0.01) between the temperature of the animals at initial care and potassium concentration, and a weak negative correlation between creatinine-related body temperature (r=−0.39; p=0.149) and BUN (r=−0.42; p=0.044). Among the laboratory parameters examined, creatinine, BUN, glucose, phosphorus, BE, HCO_3_, phosphorus, magnesium, and serum potassium demonstrated a significant decrease (p≤0.05), while ionic calcium, sodium, chloride, and blood pH exhibited significant increases (p≤0.05). There was a strong positive correlation (r=0.86; p<0.01) between potassium and creatinine variables, and a moderate correlation between potassium-related glucose (r=0.55; p=0.081) and creatinine (r=0.55; p=0.02).

Moderate or severe abnormalities were detected by ECG in 21 of 33 (63.63%) of sick felines on D1. The predominant rhythm was sinus rhythm in 18, sinoventricular rhythm in 6, sinus bradycardia in 6, and ventricular tachycardia in 3. The main abnormalities on D1 were T-waves, which peaked at 63.63% (21/33), and the same number of felines exhibited ST-segment elevation. Arrhythmias were noted in 9 of 33 (27.27%) cats and 2 of 33 (6.06%) exhibited premature ventricular complexes. On D2, T wave was observed in 7 of 33 (21.21%) and ST-segment elevation in 4 of 33 (12.12%) cats. No ECG abnormalities were detected on D7. There was a weak positive correlation between amplitude (r=0.48; p=0.014) and time (r=0.41; p=0.14) of the T wave and potassium level on D1. Eight of 33 (24.24%) cats exhibited a potassium concentration >8.5 mmol/L and ranged from 5.97 to 6.93 mmol/L in 13 (39.39%) on D1; the other animals exhibited concentrations within the reference limit.

Serial analysis of cTnI was obtained from 16 felines due to the insufficient sample to perform all tests. Serum cTnI concentrations were below the limit of detection of the assay in all the evaluations of the study, with the exception of only one feline that had concentrations significantly increased at times D1, D2, and D7 0.866 ng/mL, 0.688 ng/mL, and 0.625 ng/mL, respectively, with a very strong correlation with serum creatinine (r=0.98; p=0.10), and with serum potassium (r=0.99; p=0.055).

## Discussion

Overall, there was no significant correlation between heart rate and potassium level; however, hyperkalemia was associated with bradycardia at the time of initial care. Studies have shown that severe hyperkalemia (>8 mmol/L) is a clinical predictor of bradycardia [9]. In our study, all felines with bradycardia exhibited severe hyperkalemia. A significant increase (p<0.05) in heart rate was observed at different times, while there was a significant decrease in serum potassium concentrations, confirming the presumption that bradycardia can be attributed to hyperkalemia [9]. However, other studies [2,35] did not find such a correlation, attributing pain, and/or stress at the time of presentation to the activation of the sympathetic system and masking underlying bradycardia. Circulatory shock and accumulation of nitrogen compounds have also been reported to be associated with bradycardia and hypothermia [2,35].

There was a strong negative correlation between hypothermia and serum potassium concentration. Hypothermic patients exhibited significantly higher potassium concentrations compared to those with normothermia on D1. After clearance, hyperkalemia decreased while temperature increased significantly. A study involving 223 clogged felines reported that hypothermia was a remarkable and practical observation of the hyperkalemic state [9]. Although the results of our work linked hypothermia to hyperkalemia, other studies [2,9,35] have shown that hypothermia is related to the accumulation of uremic toxins. However, this correlation was weak in the present study. Uremic patients may have decreased T4 to T3 conversion, promoting functional hypothyroidism, triggering hypothermic conditions. Hypovolemia and cardiac dysfunction should also be considered as causes of hypothermia in these patients [9,35].

Hypotension was not observed in the animals, despite the severity of clinical changes. Other studies have also reported a lack of hypotension in patients in UO [35,36]. It has been hypothesized that acid-base and electrolyte abnormalities could have maintained pressure within the normal range in these cats. This is because there is a balance between the forces that increase and decrease blood pressure in felines and is, therefore, less susceptible to hypotension [36]. In the same study, it was also found that patients severely affected by UO had normal blood pressure, while patients who exhibited early signs of the disease were more likely to exhibit hypertension, which was also a finding in our study. Mild hypertension was evident at initial care and it was normalized on subsequent days. We may infer that specific factors contributed to hypertension on D1, such as activation of the renin-angiotensin-aldosterone system, as described in a study involving dogs with ureteral obstruction, thus increasing renin release and consequent increase in blood pressure [37]. In addition, stress and pain are also factors that have been reported to contribute to increased sympathetic nervous system activity and cause increased heart rate and blood pressure [35,36].

Metabolic acidosis was found in 18 of 33 cats (54.54%) on D1 that achieved significant normalization during treatment. Although total calcium levels remained within the reference range, there was a significant increase in subsequent days, while hyperphosphatemia normalized. Although the mean total calcium levels have remained within the reference range, seven animals presented hypocalcemia in the initial treatment. The decrease in calcium levels may be associated with the antagonistic effect of calcium on cardiotoxicity induced by high potassium concentration [9], combined with the accumulation of phosphorus. Calcium acidosis increases hyperkalemic cardiotoxicity in cats with UO and, when associated with low calcium concentrations, can further impair cardiac contractility and increase vasoconstriction [2]. Extracellular pH directly affects cell membrane excitability, reducing the availability of ß-adrenergic receptors present in cardiac nodal tissue, and it also affects cell membrane stability, promoting potassium efflux, worsening the effects of pre-existing hyperkalemia [9]. Extracellular pH directly affects cell membrane excitability, reducing the availability of ß-adrenergic receptors present in cardiac nodal tissue, and also affects cell membrane stability, promoting potassium efflux, worsening the effects of pre-existing hyperkalemia [38]. Inability of the kidneys to excrete nitrogen compounds and hydrogen ions leads to metabolic acidosis. Post-renal azotemia, hyperkalemia, and hypocalcemia exacerbate cardiotoxic damage, being supported by the strong correlation between creatinine and potassium observed in this study.

Hyperglycemia has been reported in studies involving cats with UO [9,35]. One study reported significantly higher glycemia in cats that were clogged for >36 h compared with those clogged for <36 h [35]. Another investigation reported hyperglycemia in 54% of felines obstructed at admission [9], but none of the studies demonstrated a probable relationship between hyperglycemia and serum creatinine and potassium levels, as observed in this study. Moreover, hyperglycemia may be used as an indicator of the severity of hyperkalemia and azotemia in cats with UO. It is noteworthy that other factors, such as pain and stress, can raise blood glucose levels and should, therefore, be considered as differential diagnoses [9,35].

Magnesium is fundamental for many bodily functions, including intracellular signaling, oxidative phosphorylation, cardiovascular tone, and neuromuscular excitability [39]. Hypermagnesemia observed in the early stages is due to acute kidney injury (AKI) from UO. Although asymptomatic, worsening of the cardiac effects of hyperkalemia can be observed, resulting in atrioventricular block, PR interval prolongation, and bradycardia [39,40], which were not documented in this study due to the relative elevation of this ion in the studied patients. Sodium and chloride concentrations in our study were within the normal range, despite a significant increase after the clearance procedure. This fact largely reflects the decreased food intake and gastrointestinal losses caused by the disease [41].

Myocardial dysfunction is common in critically ill patients and it has been associated with high mortality rates in humans [18,20,42]. Studies involving dogs have reported similar results, even without clinical alterations [43,44]. The presence of arrhythmias and murmurs in felines with UO during hospitalization has been reported [21]. Although our study did not reveal the presence of murmurs on cardiac auscultation during the follow-up period, ECG abnormalities were observed in 63.63% of the felines. Arrhythmias, flat P waves, apical T waves, ST-segment alteration, and premature ventricular complexes were observed on D1, with maintenance of T wave and ST segment changes in some patients after 24 h. ECG abnormalities in obstructed felines are mainly due to AKI-induced metabolic disturbances, which may lead to severe effects on myocardial activity and function [45,46]. AKI induces pro-inflammatory cytokines and activates the sympathetic nervous and renin-angiotensin-aldosterone systems and may increase the risk for cardiac injury; therefore, long-term ECG monitoring is generally indicated in these patients [45].

Although cats developed arrhythmias and had azotemia and hyperkalemia, we were unable to detect changes in cTnI concentrations in 93.75% (15/16) animals studied, suggesting that myocardial damage was mild at most of them and it did not cause clinically relevant cardiac dysfunction. Interestingly, the previous studies have reported an increase in troponins in dogs and cats without heart disease and with AKI [23,28,45]. To date, only one study has evaluated cTnI concentrations in three cats with UO, although high, the authors reported that two of the three cats studied showed evidence of chronic kidney disease [28]. Although our study did not assess the presence of underlying diseases in the animals studied, the presence of high concentrations of cTnI in a feline, with a gradual decrease in the different times evaluated, demonstrated a very strong correlation (r=0.98 and r=0.99) with the concentration of serum creatinine and potassium, respectively. It can be inferred that severe azotemia and hyperkalemia contributed to the evidence of myocardial injury even without relevant electrocardiographic manifestations. However, comorbid conditions, such as hidden heart disease, may have been responsible, at least in part, for the elevated troponin concentration in this feline.

## Conclusion

Monitoring felines with UO demonstrated that cardiovascular changes occur concomitantly with metabolic changes and are improved after stabilization of the condition. In addition, our results demonstrated that the sum and severity of electrolyte abnormalities, such as metabolic acidosis, hyperkalemia, hyperglycemia, hyperphosphatemia, and hypocalcemia, aggravate the clinical and cardiovascular status of these patients. Another important point is that hyperglycemia in felines with UO appears to be associated with decreased renal clearance, which may reflect the severity of hyperkalemia and azotemia. However, cTnI, blood pressure, and heart rate within the reference range do not exclude the presence of major cardiovascular, electrolyte, and acid-base abnormalities that require immediate correction. We were able to identify and monitor metabolic changes that may induce severe cardiovascular impairment in cats with UO.

## Authors’ Contributions

DHC and ABPFA: Contributed to the conception and design of the study. DHC: Wrote this manuscript. DHC, MEP, MNF, and YPAT: Contributed to data collection. CZ and AJM: Participated in laboratory analysis of the samples. DHC, VRFS, JB, and ABPFA: Performed analytical work. DHC, VRFS, and ABPFA: Critically revised the manuscript. All authors read and approved the final manuscript.
